# Impact of carbonization conditions and adsorbate nature on the performance of activated carbon in water treatment

**DOI:** 10.1186/s13065-023-01091-1

**Published:** 2023-11-22

**Authors:** Ibrahim Karume, Simon Bbumba, Simon Tewolde, Is’harq Z. T. Mukasa, Muhammad Ntale

**Affiliations:** 1 Department of Chemistry, College of Natural Sciences, Makerere University, P. O. Box 7062, Kampala, Uganda; 2 Department of Chemistry, Faculty of Science, Kabale University, Kabale, Uganda

**Keywords:** Temperature, Activators, Surface properties, Adsorption capacity, Adsorbate nature

## Abstract

The physical and chemical structure of activated carbon (AC) varies with the carbonization temperature, activation process and time. The texture and toughness of the starting raw material also determine the morphology of AC produced. The Brunauer-Emmet-Teller surface area (S_BET_) is small for AC produced at low temperatures but increases from 500 to 700 °C, and generally drops in activated carbons synthesized > 700 °C. Mild chemical activators and low activator concentrations tend to generate AC with high S_BET_ compared to strong and concentrated oxidizing chemicals, acids and bases. Activated carbon from soft starting materials such as cereals and mushrooms have larger S_BET_ approximately twice that of tough materials such as stem berks, shells and bones. The residual functional groups observed in AC vary widely with the starting material and tend to reduce under extreme carbonization temperatures and the use of highly concentrated chemical activators. Further, the adsorption capacity of AC shows dependency on the size of the adsorbate where large organic molecules such as methylene blue are highly adsorbed compared to relatively small adsorbates such as phenol and metal ions. Adsorption also varies with adsorbate concentration, temperature and other matrix parameters.

## Introduction

Human population growth accompanied by economic and industrial developments continuously demand clean and fresh water supplies. Manufacturing, mining and agriculture release pollutants with detrimental effects on man and the environment. Heavy metals in water have toxic effects resulting in death and chronic illness. Reports show a cancer risk of ~ 76% and ~ 15.7% was caused by cadmium and arsenic, respectively from the adsorption of the metals from the soil in paddy rice [1]. Most hazardous non-essential heavy metals and metalloids, for example, arsenic, lead, cadmium, and mercury have been reported in food crops and are deleterious in various respects [2, 3]. Oil exploration sites register the highest pollutants [4, 5]. There are also health hazards of fertilizers, pesticides, antibiotics and dyes from industrial discharge [6]. Therefore, it is crucial to find an environmentally benign approach to curb water pollution. Common water treatment techniques include; magnetic separation [7], flocculation [8], filtration [9], reverse osmosis [10] and adsorption [11, 12]. Physicochemical treatments that involve coagulation-flocculation processes were generally found to be unable to remove pharmaceuticals and personal care products [13].

Adsorption processes are generally cheap, easy to operate, and highly efficient. During an adsorption-based wastewater treatment process, organic and inorganic pollutants (adsorbates) are attracted to the outer and inner surfaces of the porous materials (adsorbents), through mass transfer and diffusion from the aqueous media to the active adsorbing sites of the adsorbents. The mechanisms are either chemisorption involving ionic interactions or physisorption such as Van der Waals and π–π interactions or both [14–16]. Several materials such as clays, zeolites and carbon have been used as adsorbents but the structure of the latter (carbon) can be predicted and tuned to meet specific adsorption needs where a target pollutant can be removed efficiently.

**Fig. 1:**
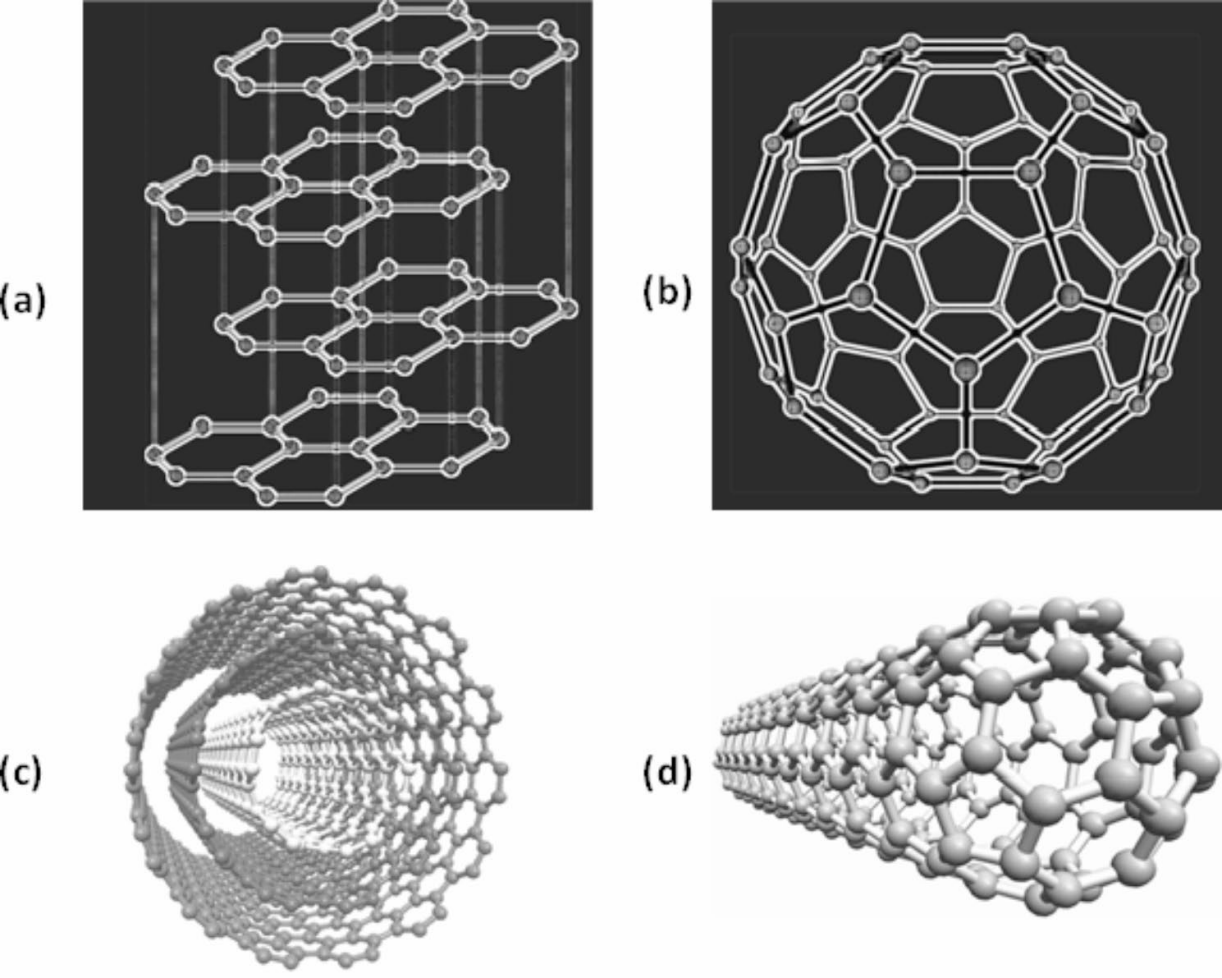
(**a**) Graphite made of layers of graphene; (**b**) Fullerene made of graphene layer joined end to end; (**c**) Multi-walled carbon nanotube with concentric hollow graphene rings; (**d**) Single-walled carbon nanotube with a half-fullerene endcap [19]

Carbon with four electrons to complete its octet gives it unique properties among which the ability to form multiple bonds with the saturation of electrons like in the case of the diamond where it is a non-conductor of electricity or a more interesting arrangement with unsaturation and hence free electrons that enable electrical conductivity in graphite and amorphous carbon. Perhaps the other unique feature is the ability of carbon atoms to bond with an irregular fashion in charcoal or a regular packing to form crystals, for instance, the graphitic form such as fullerenes first discovered by Kroto [17], graphene and carbon nanotubes first synthesized by Iijima [18] which can have open ends or one end capped with half fullerene (Fig. 1).

**Fig. 2:**
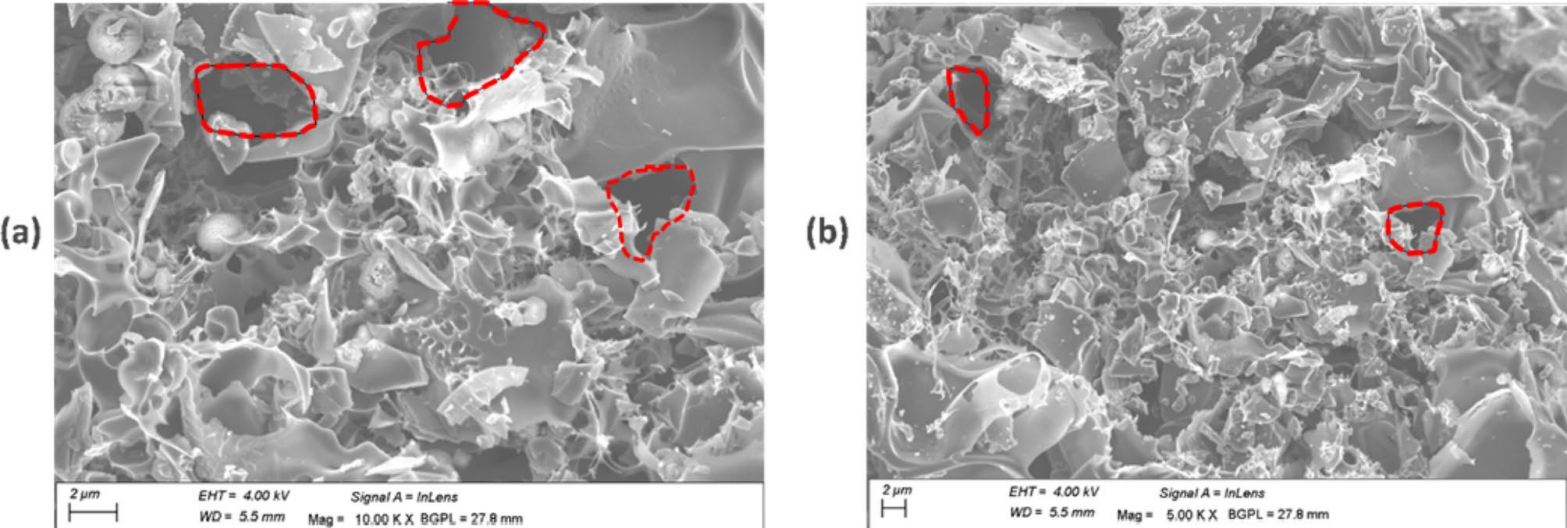
SEM image showing varying surface pore sizes (indicated by red dotted lines) in activated carbon produced from banana peels carbonized at: (**a**) 600 °C; (**b**) 700 °C [22]

The hollow and enclosed nature of crystalline carbon has versatile applications in material storage, for instance, hydrogen [20]. Electric charge storage where carbon nanomaterial from plastic wastes was used to make supercapacitors has been reported [21]. Perhaps the most interesting is the amorphous form of carbon which despite the irregular arrangement tend to have carbon atoms stuck together with crevices and internal micropores in the structure where both organic and inorganic compounds in liquid and gas phase can be stored via physical and chemical linkages which is enhanced by the presence of other functional groups especially for activated carbon derived from biomass. Activated carbon has a large surface area and creates voids of varying pore volumes depending on the raw material carbonized. This is visible as dark spots in images from scanning electron microscopy (SEM) (Fig. 2). Microporosity of carbon has been utilized in water purification and the removal of organic compounds such as dye in wastewater from textile industries.

Perhaps the most interesting is the existence of heteroatoms that remain after carbonization. These result from the action of heat or chemical interaction of chemical activators on functional groups of building parts of plants or animals. Heteroatoms enable chemisorption which not only enhances the adsorption capacity but also increase the range of adsorbates. It is against this background that we envisaged reviewing the correlation of the structural properties of activated carbon to the carbonization conditions and relating it to the nature of the starting raw material and the adsorption capacity.

## Structural properties of activated carbon based on the source of carbonized material and conditions

Surface properties of activated carbon such as surface area, pore size and pore volume are essential and an indication of the extent of heterogeneity of AC and give a clue of its internal structure [23], which highly depends on the method of activation and synthesis [24]. Synthesis of activated carbon involves two stages; first, carbonization which involves the thermal decomposition of raw materials under an inert atmosphere to remove functional groups containing heteroatoms resulting in the production of biochar. The second stage is activation which can be by physical reagents using selected gases such as steam and carbon dioxide or chemical activation where oxidizing and dehydrating chemicals are employed [25–27]. Activation increases the surface area of biochar which is usually below 300 m^2^/g after carbonization attributed to clogged pores by tarry materials [28, 29]. However, the extended time during activation at high temperatures tends to lower the yield of AC [30]. The decrease in yield at high temperatures is attributed to the secondary decomposition of biochar but it improves the quality of AC [31]. Nevertheless, chemical activation combines the activation and carbonization processes saving heat energy and hence low temperatures required. It however requires a cleaning process to flush the unreacted chemicals and undesired products [32, 33]. Commonly used chemical activators include ZnCl_2_, KOH, NaOH, H_2_SO_4_ and H_3_PO_4_. Chemical activation induces cyclization, dehydration and condensation reactions that promote pyrolytic decomposition and formation of cross-linkages which aid in the creation of both mesopores and micropores [34, 35].

This section summarizes how the structural properties of activated carbon such as surface area, pore size and volume, and residual functional groups present in AC relate to the raw material carbonized, the temperature of carbonization and the chemical activator used. Further, the dependence of the adsorption capacity on the nature of the adsorbate and S_BET_ was also investigated.

### Surface area of activated carbon from soft and hard raw materials

**Fig. 3:**
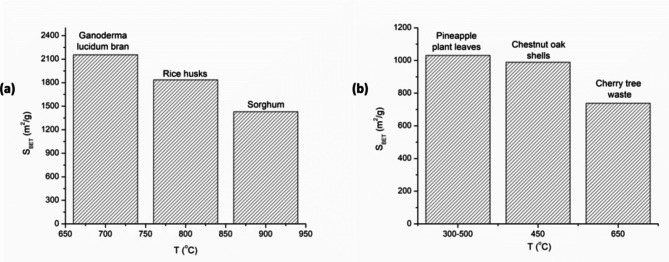
Variation of BET surface area with the nature of carbonized material; (**a**) Soft materials; (**b**) Tough materials. The data used to generate the graphs was obtained from the literature (Table 1)

The surface area of activated carbon is highly dependent on the softness/hardness of the material to be carbonized (Table 1). Generally, tough materials such as stem barks, bone and shells yield activated carbon with small S_BET_ compared to soft and tender raw materials such as leaves, cereals and soft plants such as mushrooms. This is evident from the relatively small S_BET_ (316–989 m^2^/g)) for activated carbon derived for instance, from chicken bone (316 m^2^/g) [36],, black wattle bark (414 m^2^/g) [37], sugarcane bagasse (692 m^2^/g) [38] and chestnut oak shells (989 m^2^_/_g) [39] as compared to S_BET_ > 1000 m^2^/g for activated carbon synthesized from rice husks, sorghum and *Ganoderma lucidum* [40–42] which is likely due to the ease for heat penetration that burns the soft materials uniformly (Fig. 3). It is also worth noting that solid lamps such as bones, stem barks and shells have a reduced surface area compared to grains like sorghum and rice husk pellets which might have contributed to the low S_BET_ observed in AC of the latter and vice versa.

**Table 1:** Variation of BET surface area, total pore volume and residual heteroatom with carbonization conditions

Raw material	T. and t.	Activator	Functional groups	Vt (cm3/g)	SBET (m2/g)	Application in water treatment	Reference
Palm kernel shell	500°C, 2 h	H_3_PO_4_	O-H, N?H, C = O, C = C, C?O?C, C?O?H, HC = CH	-	-	Treatment of greywater	[43]
Chicken bone	500°C, 2 h	KMnO_4_	O-H, C = O, PO_4_^3-^	0.007	316	Removal of tetracycline and fluorescence dye	[36]
Leucaena leucocephala biomass	800°C, 1.5 h	NaOH, N_2_	O-H, C-O, C = O	-	776	Removal of cadmium	[44]
Sorghum	900°C, 1.5 h	KOH, N_2_	O-H, C-H, C-O, C = O	0.784	1430	Adsorption of methylene blue	[41]
Sugarcane bagasse	800°C, 1 h	KOH, N_2_	O-H, C-C, C-H, C = C, C-O, C = O	0.5	692	Removal of naphthalene	[38]
Vitis vinifera leaf litter	600°C, 1 h	ZnCl_2_, H_3_PO_4_, N_2_	C-H, C = O, C = C	0.03	617	Adsorption of phenanthrene	[45]
Coffee waste	350°C, 3.5 h	HCl	O-H, N-H, C-H	-	-	Treatment of nitrate and nitrite fertilizers in industrial wastewater	[46]
Fruit shells	400°C, 1 h	N_2_	O-H, C = O, C-H, C-O, C-C, C = C	0.235	579	Adsorption of methylene blue and ofloxacin	[47]
Hide waste	700°C, 1 h	K_2_SiO_3_	O-H, C-H, C-O, C = O, C = C, N-H, Si-N	-	1804	Removal of nickel (II) ions	[48]
High-pressure steaming hides waste	700°C, 1 h	K_2_SiO_3_	O-H, C-H, C-O, C = O, C = C, N-H, Si-N	-	1361	Removal of nickel (II) ions from water	[48]
Pineapple plant leaves	300°C, 1 h then 500°C, 1 h	H_3_PO_4_, N_2_	O-H, C-H, C = O, C-O	1.270^a-fibres^	1031	Adsorption of caffeine	[49]
Chestnut oak shells	450°C, 2.5 h	H_3_PO_4_, N_2_	O-H, C-H, C-O, C = O	0.71	989	Removal of chromium (VI)	[39]
Rice husks	800°C, 2 h	KOH, CO_2_	O-H, C-H, C = O, C = C, C ≡ C	1.126	1836	Removal of phenol	[40]
Date stones	660 W MV radiation, 8 min	K_2_CO_3_	O-H, C-C, C-O, C-X	0.656	1144	Adsorption of methylene blue	[50]
Cherry tree waste	650°C, 2 h	H_3_PO_4_,	O-H, C-H, C-O, C = O, C = C	0.558	738	Adsorption of cationic red 14 dye	[42]
Black wattle bark waste	500°C for 2 h 700°C, 4 h	ZnCl_2_	O-H, C-H, C = O	0.064	414	Adsorption of phenol	[37]
Ganoderma lucidum bran	300°C, 3 h	ZnCl_2_, N_2_	O-H, C-H, C = C, C-O	0.03	18	Adsorption of copper ions	[51]
Ganoderma lucidum bran	300°C, 3 h	KMnO_4_, N_2_	O-H, C-H, C = C, C-O	0.11	832	Adsorption of copper ions	[51]

Unless stated, all the data is from peer-reviewed research articles referenced

It can be observed that irrespective of the raw material being carbonized, S_BET_ decreased with increased temperature and it is relatively low when base activators are used (Table 1). There is no clear correlation between total pore volume and any of the carbonization conditions, however, the pore volume generally increases with an increase in the BET surface area.

### Effect of temperature, activator and time of carbonization on total pore volume and surface area of activated carbon

**Fig. 4:**
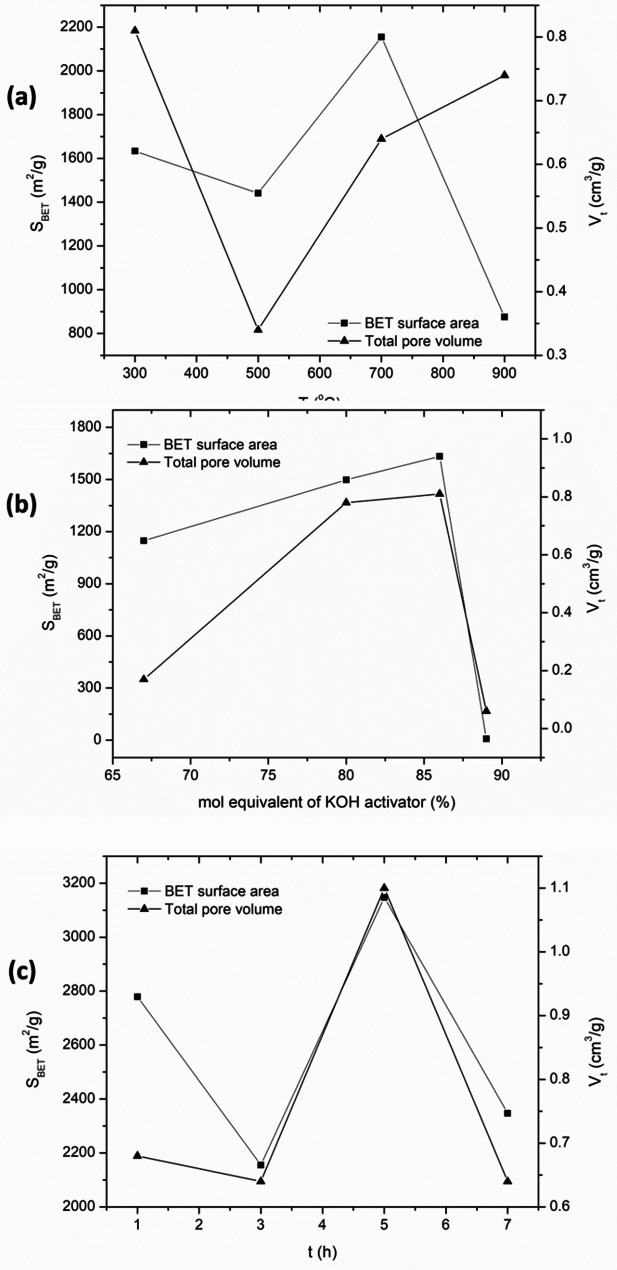
Dependency of BET surface area and total pore volume of activated carbon on: (**a**) Carbonization temperature; (**b**) Concentration of chemical activator; (**c**) Carbonization time. The data used to generate the curves was obtained from recent research findings [51]

The properties of activated carbon largely depend on the mode of activation and the carbonization temperature. Physical activation employs oxidizing gases like air, carbon dioxide and steam at higher temperatures that imparts pores on the surface of the carbonized material which increases the surface area and enhances the porosity [52]. On the other hand, chemical activation by acidic and basic activators or other oxidizing agents such as potassium permanganate dehydrate or oxidize the raw material of plant or animal origin thereby reducing the moisture content and also altering functional groups which would otherwise be difficult to eliminate during the heating. This leaves mostly the carbon content thus a further increase of the surface area. It is worth noting that activated carbon with small S_BET_ is produced at both low concentrations of the activating agent and low temperature and S_BET_ increases on an increase in temperature and activator concentration but to a certain limit. Extreme temperatures, long heating times and exceedingly higher proportions of the activator tend to reduce the S_BET_ as observed from a study by Wang and group [51] (Fig. 4).

**Fig. 5:**
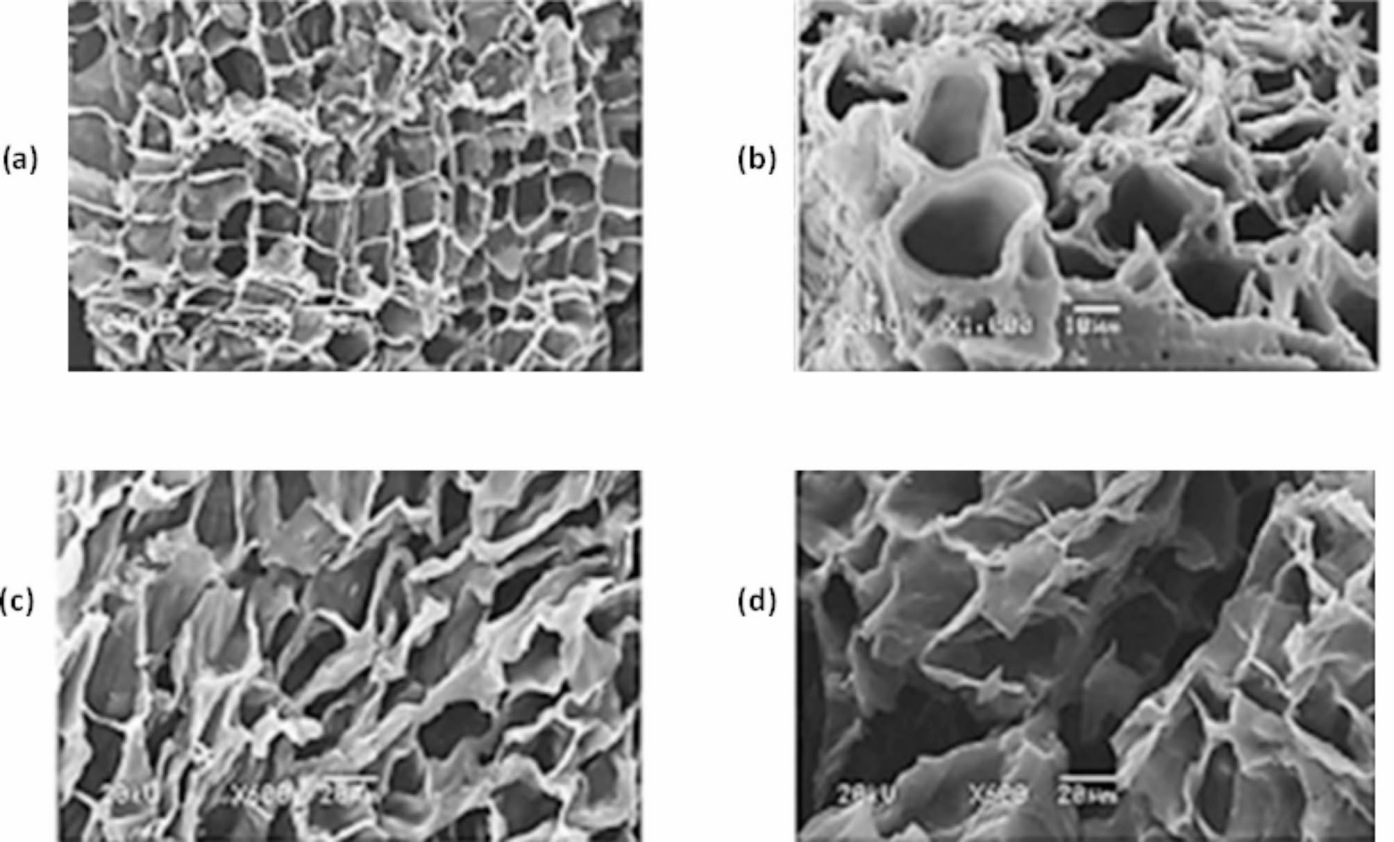
SEM images of activated carbons prepared from paulownia wood chemically activated using different mass of phosphoric acid to wood powder ratios at 400 °C: (**a**) 1:1; (**b**) 2:1; (**c**) 3:1; (**d**) 4:1 [53]. Reprinted with permission from Elsevier License Number: 5,601,360,125,916

**Fig. 6:**
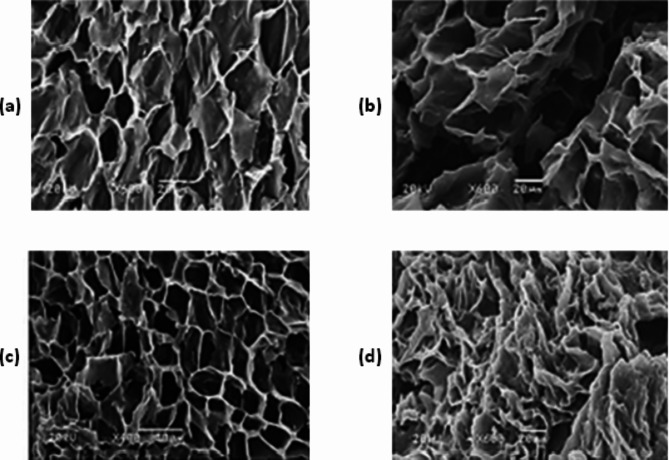
SEM images of activated carbons prepared from paulownia wood carbonized at different temperatures: (**a**) 300 °C; (**b**) 400 °C; (**c**) 500 °C; (**d**) 600 °C [53]. Reprinted with permission from Elsevier License Number: 5,601,360,125,916

The surface texture, pore shape and pore size also vary greatly with carbonization temperature and the concentration of activator used during production of AC. This can be observed of SEM images of AC synthesized from paulownia wood (Figs. 5 and 6).

It is worth to tell that physical activation significantly affects the structural properties of activated carbon. Higher surface area and larger total pore volume and pore sizes was observed in activated carbon synthesized by chemical activation using potassium hydroxide compared to the one prepared by two-activation process using carbon dioxide as the physical activator in addition to the base (1836 vs. 1628 m^2^/g, 1.126 vs. 0.999 cm^3^/g and 0.258 vs. 0.150 cm^3^/g for mesopores/ 0.805 vs. 0.648 cm^3^/g for micropores, respectively) [40].

### Effect of carbonization conditions on residual functional groups present in activated carbon

Besides the large surface area and pores, the presence of residual functional groups enhances the adsorption capacity of activated carbon. Hydrogen and electronegative atoms are left after volatilization by heat, and chemical oxidation and other reactions by chemical activators on groups such as phenols, carboxylic acids, alcohols, amines etc. The presence of heteroatoms in activated carbon allows interactions such as electrostatic attractions of metals and hydrogen bonding with pharmaceuticals and other organic pollutants. Therefore, the presence of other atoms/functional groups in activated carbon is vital.

**Fig. 7:**
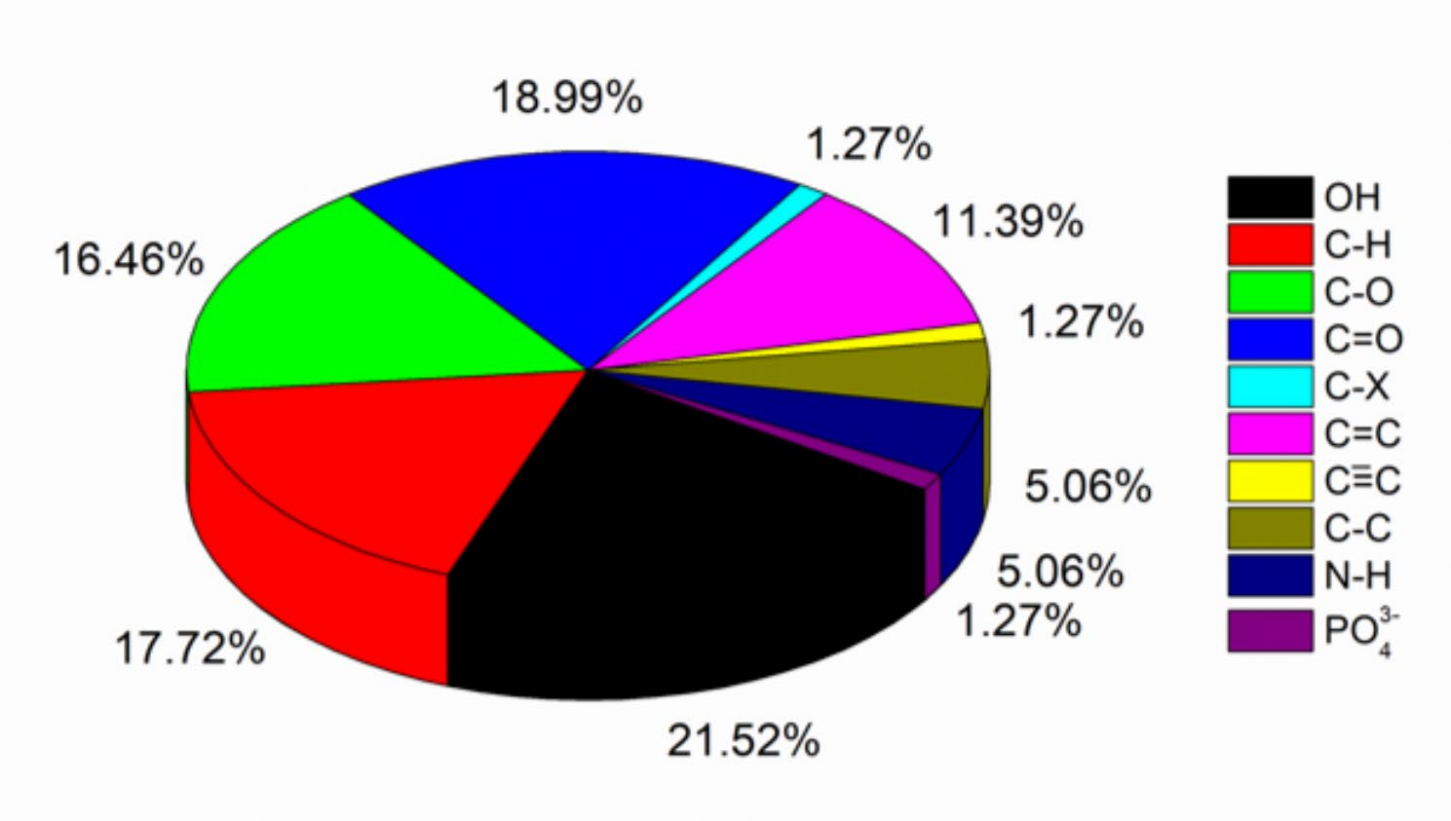
Residual function groups in activated carbon. The data used to generate the graphs was obtained from the literature (Table 1)

Carbonization conditions especially the temperature and the chemical activator determine the number and nature of functional groups present in activated carbon. The residual hydroxyl stretch is observed in the infrared spectra of almost all synthesized activated carbons except in one instance where a combination of zinc chloride and phosphoric acid were used as chemical activators [45]. This suggests a destructive effect of chemical activators on the functional groups present in the raw material before carbonization. The presence of residual heteroatoms is summarized in Fig. 7.

**Fig. 8:**
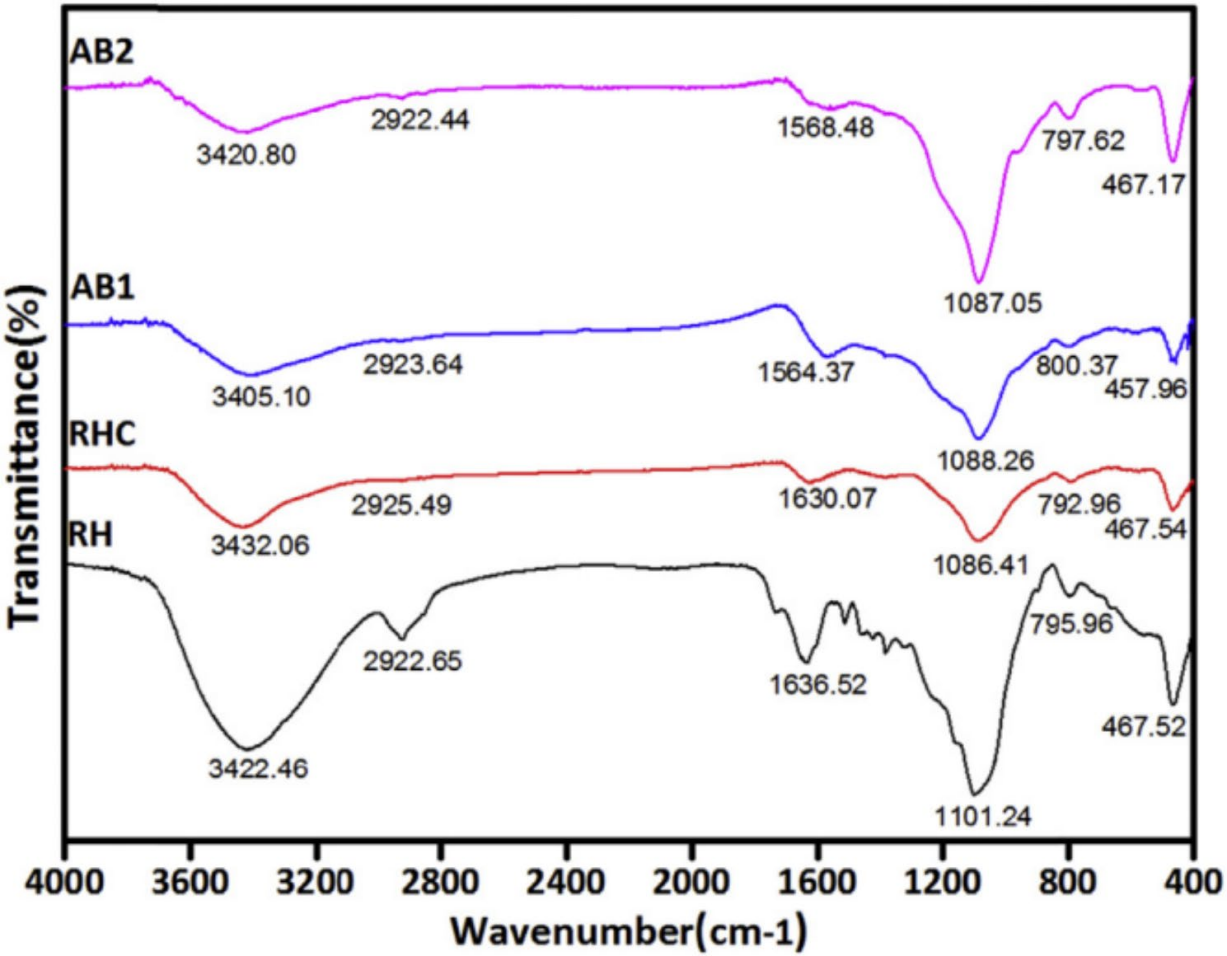
Infrared spectra of rice husks (RH), rice husks char (RHC) and carbon activated by: KOH only (AB1 ) and KOH/CO_2_ (AB2). Reprinted with permission from Elsevier License Number: 5,658,041,104,498

The number of heteroatoms present largely depends on the nature of the starting material, however, their presence in the carbonized material is reduced with an increase in temperature and the oxidizing potential of the activator (Table 1). High heats provide sufficient energy to break chemical bonds, a similar scenario caused by strong oxidizing agents. Nevertheless, the absence of a chemical activator [47] or the use of mild activators such as potassium silicate tends to leave many heteroatoms even at high carbonization temperatures [48]. Generally, heating biomass to carbonaceous materials and their surface activation eliminates the functional groups present in the raw material. However, activation does not alter much heteroatoms present as seen infrared spectra (Fig. 8) of rice husks and the formed char and activated carbon as reported by Yafei and Yuhong [40].

## Dependency adsorption capacity of activated carbon on its morphology and the nature of adsorbates

Activated carbon is mostly used in water purification and other contaminated liquids to remove unwanted pollutants [25, 54]. Adsorption depends on the extent of porosity and the electrostatic interactions which indicates dependency on the nature of the adsorbates since they have varying functional groups and sizes that influence the interaction with the carbonaceous materials and the leaching process (desorption), respectively.

The adsorption capacity of activated carbon depends on the nature adsorbate and S_BET_ of the AC (Table 2). For a similar class of compounds, it can be observed that metal ions are weakly adsorbed (< 50 mg/g) compared to organic compounds. In the reported research works, adsorption capacities of 35 mg/g and 33 mg/g of chromium(VI) and nickel(II) ions, respectively were achieved despite the large S_BET_ of AC used [39, 48]. Structural properties; S_BET_ and V_t_ play a great role in adsorption as observed from the exceedingly high adsorption capacity of methylene blue (935 mg/g) using AC with large S_BET_ (1430 m^2^/g) [41] compared to 199 mg/g by AC with S_BET_ 579 m^2^/g [47].

**Table 2:** Dependence of adsorption capacity of AC on S_BET_ and nature of the adsorbate

Adsorption capacity (mg/g)	S_BET_ (m^2^/g)	Adsorbed material	Reference
935	1430	Methylene blue	[41]
199	579	methylene blue & ofloxacin	[47]
35	1804	Nickel(II)	[48]
156	1031	Caffeine	[49]
33	989	Chromium(VI)	[39]

**Fig. 9:**
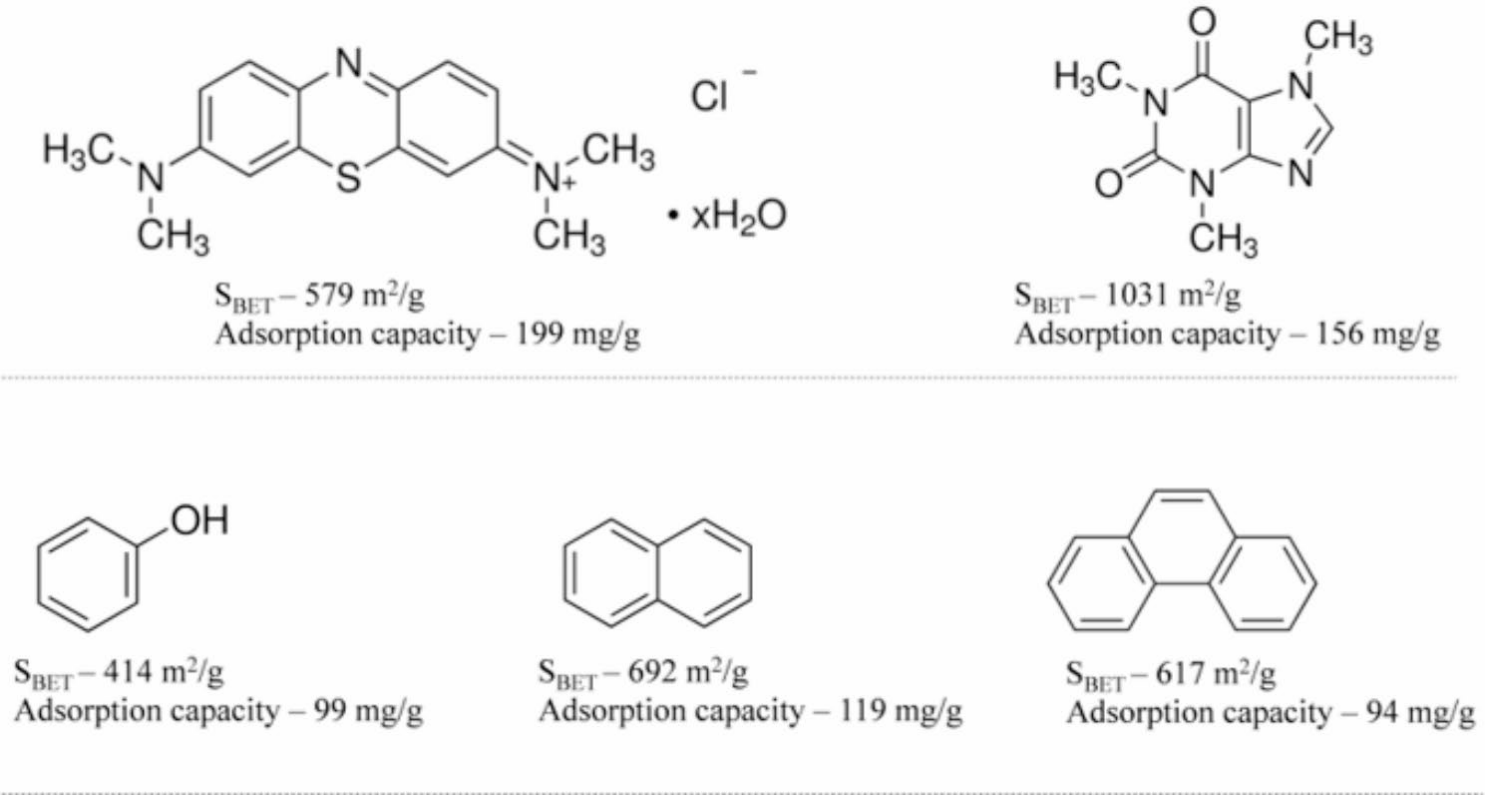
The adsorption capacity of organic compounds of varying sizes

Further, it can also be observed that large molecules are highly adsorbed compared to small adsorbates. Similar to the poor adsorption of the relatively small metal ions, it can be seen that larger organic compounds are highly adsorbed by AC with S_BET_ in the range 400–700 m^2^/g, for instance, methylene blue (199 mg/g) [47] compared to ≤ 119 mg/g for relatively smaller organic adsorbates for example, phenol, naphthalene and phenanthrene [37, 38, 45] (Fig. 9).

**Fig. 10:**
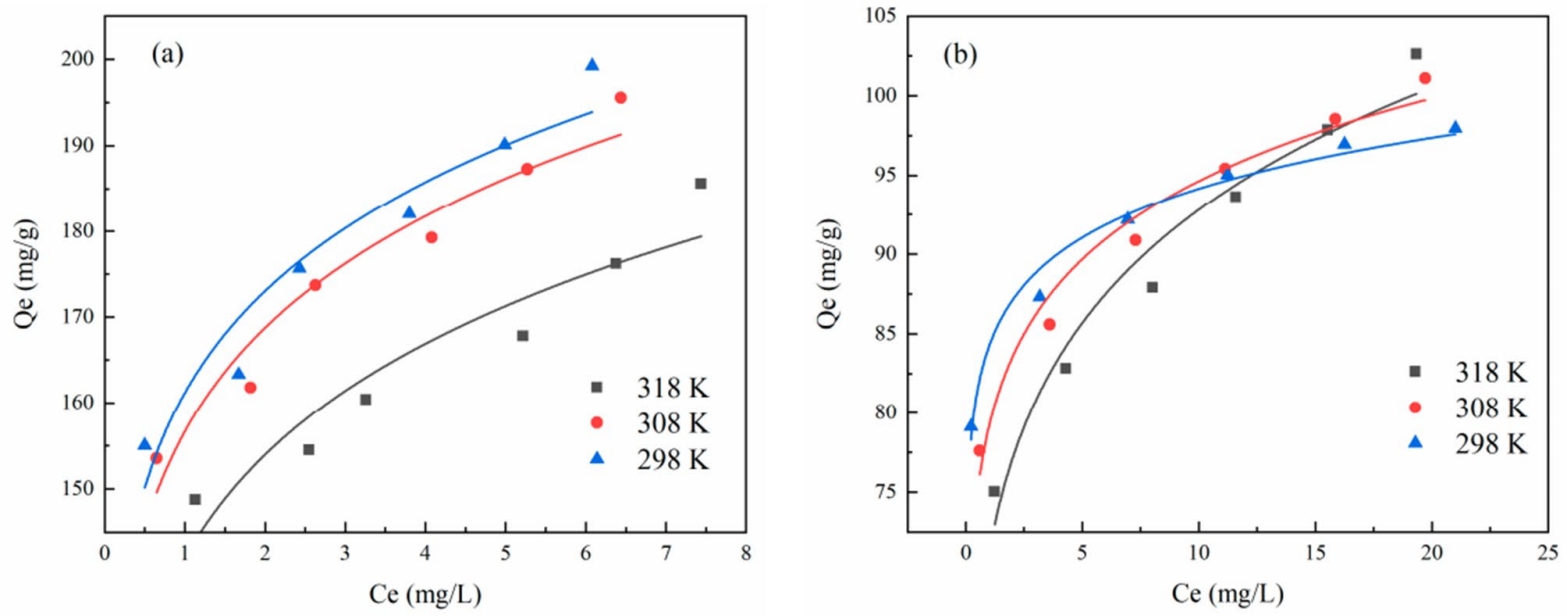
Effect of concentration and temperature on adsorption methylene blue (**a**) and ofloxacin (**b**) by fruit-shell activated carbon [47]

The adsorption behaviour of activated carbon is also highly dependent on the porosity of the material and the adsorbate concentration. At relatively low adsorbate concentration, activated carbon with large pore sizes/volume showed a higher edge to one with relatively small pore sizes and volume. Nevertheless, the performance of both materials was similar with the former having a slight edge which likely due to slow diffusion/leaching of the adsorbed material out of the AC with larger pore volume [40]. The increase in adsorption capacity with an increase in adsorbate concentration was also observed in work reported by Jinlong et al. however, poor adsorption of both methylene blue and ofloxacin was recorded with increasing temperature at low adsorbate concentrations (Fig. 10) [47]. On the contra, at higher adsorbate concentration, enhanced adsorption of ofloxacin was observed at low temperatures which suggests adsorbate-adsorbent interactions dependent on the nature of the adsorbate species since they are made of different chemical composition.

## Conclusions

The carbonization conditions and nature of starting material determine the morphology of activated carbon. Soft materials tend to give AC with large S_BET_. Extremely high carbonization temperatures (> 700 °C) and strong chemical activators at high concentrations reduce S_BET_ and also eliminate heteroatoms thereby reducing the residual function groups. For enhanced adsorption capacities of AC, soft materials should be carbonized at relatively low temperatures (< 600 °C) and vice versa. Large organic compounds are highly adsorbed compared to small adsorbates like metal ions. The porosity of carbon influences the diffusion and leaching of materials in and out, respectively. Adsorption performance of AC is also highly dependent on the adsorbate particles present in water, temperature and other physical parameters.

## Data Availability

Not applicable.
